# Independent Pre-Transplant Recipient Cancer Risk Factors after Kidney Transplantation and the Utility of G-Chart Analysis for Clinical Process Control

**DOI:** 10.1371/journal.pone.0158732

**Published:** 2016-07-11

**Authors:** Harald Schrem, Valentin Schneider, Marlene Kurok, Alon Goldis, Maren Dreier, Alexander Kaltenborn, Wilfried Gwinner, Marc Barthold, Jan Liebeneiner, Markus Winny, Jürgen Klempnauer, Moritz Kleine

**Affiliations:** 1 General, Visceral and Transplant Surgery, Hannover Medical School, Hannover, Germany; 2 Core Facility Quality Management & Health Technology Assessment in Transplantation, Integrated Research and Treatment Center Transplantation (IFB-Tx), Hannover Medical School, Hannover, Germany; 3 Gynecology and Obstetrics, KRH Klinikum Nordstadt, Hannover, Germany; 4 Lean Six Sigma Black Belt, Amstelveen, The Netherlands; 5 Institute of Epidemiology, Social Medicine and Health Systems Research, Hannover Medical School, Hannover, Germany; 6 Trauma and Orthopedic Surgery, Federal Armed Forces Hospital Westerstede, Medical Service of the Federal Armed Forces, Westerstede, Germany; 7 Nephrology, Hannover Medical School, Hannover, Germany; Taipei Medical University, TAIWAN

## Abstract

**Background:**

The aim of this study is to identify independent pre-transplant cancer risk factors after kidney transplantation and to assess the utility of G-chart analysis for clinical process control. This may contribute to the improvement of cancer surveillance processes in individual transplant centers.

**Patients and Methods:**

1655 patients after kidney transplantation at our institution with a total of 9,425 person-years of follow-up were compared retrospectively to the general German population using site-specific standardized-incidence-ratios (SIRs) of observed malignancies. Risk-adjusted multivariable Cox regression was used to identify independent pre-transplant cancer risk factors. G-chart analysis was applied to determine relevant differences in the frequency of cancer occurrences.

**Results:**

Cancer incidence rates were almost three times higher as compared to the matched general population (SIR = 2.75; 95%-CI: 2.33–3.21). Significantly increased SIRs were observed for renal cell carcinoma (SIR = 22.46), post-transplant lymphoproliferative disorder (SIR = 8.36), prostate cancer (SIR = 2.22), bladder cancer (SIR = 3.24), thyroid cancer (SIR = 10.13) and melanoma (SIR = 3.08). Independent pre-transplant risk factors for cancer-free survival were age <52.3 years (p = 0.007, Hazard ratio (HR): 0.82), age >62.6 years (p = 0.001, HR: 1.29), polycystic kidney disease other than autosomal dominant polycystic kidney disease (ADPKD) (p = 0.001, HR: 0.68), high body mass index in kg/m^2^ (p<0.001, HR: 1.04), ADPKD (p = 0.008, HR: 1.26) and diabetic nephropathy (p = 0.004, HR = 1.51). G-chart analysis identified relevant changes in the detection rates of cancer during aftercare with no significant relation to identified risk factors for cancer-free survival (p<0.05).

**Conclusions:**

Risk-adapted cancer surveillance combined with prospective G-chart analysis likely improves cancer surveillance schemes by adapting processes to identified risk factors and by using G-chart alarm signals to trigger Kaizen events and audits for root-cause analysis of relevant detection rate changes. Further, comparative G-chart analysis would enable benchmarking of cancer surveillance processes between centers.

## Introduction

Kidney transplantation has become the preferred treatment option for patients with renal failure since the first successful clinical transplantation in 1954 [1, 2]. The success story of kidney transplantation led to a steadily increasing patient and graft survival [2]. De novo malignancy is one of the leading causes of early death after solid organ transplantation and a rising number of reports on de novo malignancy after renal transplantation were published [3–30], with identified methodological weaknesses such as the failure to provide the total person-years at risk or to define inclusion and exclusion criteria [31]. The rising number of long-term survivors after transplantation puts long-term complications and their management more and more into the focus of attention.

Long-term cancer risk is known to vary significantly depending on ethnicity, demographic variations and geographical epidemiological differences in the prevalence of viral diseases with associated increased cancer risks [32]. The determination of comparative cancer risks after transplantation faces the methodological challenges associated with differing inclusion and exclusion criteria for some cancer types, pediatric cases, different minimal post-transplant observation and survival times, various transplant indications that may be associated with different cancer risks and variations in immunosuppressive regimens [21, 23, 32]. It is therefore no surprise that only a few investigations with age- and sex-matched control populations from different regions of the world are available so far [11, 23, 26–27, 29–30]. There are no studies available so far that identify independent pre-transplant risk factors for de novo cancer in Germany.

G-charts are based on the geometric distribution and were designed to monitor rare events. In health care they have initially been developed to monitor and illustrate cardiac bypass infections, catheter-associated infections, surgical site infections, contaminated needle sticks, osteomyelitis treatment failures and medication errors [33–35]. While conventional charts often result in subgroups being plotted too infrequently for real-time control of these problems, particularly when dealing with infrequent events or low “defect” rates, G-chart analysis is based on inverse sampling to detect process changes or verify improvements faster [33, 34]. Prospective G-chart analysis is able to trigger specific awareness when relevant increases or decreases of rare events are detected. Such alarms enable timely root cause analysis to secure early clinical process improvement [33, 34, 36].

The current study determines site-specific cancer incidence rates in patients after kidney transplantation compared to an age- and sex-matched general reference population in Germany and characterizes independent pre-transplant risk factors for cancer-free survival with possible implications for individualized long-term cancer surveillance. Findings are used to explore the principal suitability of G-chart analysis to identify relevant changes in cancer detection rates with the goal to develop a strategy for improvement of aftercare.

## Patients and Methods

### Inclusion and exclusion criteria

All consecutive kidney transplants from the 01.01.2000 until the 31.12.2012 with follow-up in our adult outpatient clinic were analyzed. We did not exclude 40 pediatric cases who have reached age 18 years prior to their first follow-up visit after transplantation in our adult transplant outpatient clinic because we feel that they were managed exactly the same way as all other adult patients. Combined transplants, re-transplants, and cases with 30-day post-transplant mortality were excluded. Non-melanoma skin cancers were not analyzed since they are not registered in cancer registries.

### Data collection and interpretation

This is a retrospective single-center analysis with 9,425 person-years of follow-up until the 18.02.2014 (mean follow-up 5.7 years, median 5.1 years, and range: 0.1–14.0 years) with observational endpoints being either first diagnosis of malignancy, death, graft failure or the date patients were last seen alive. All patients were followed in our outpatient transplant clinic as described before [32].

### Ethics statement

According to the Professional Code of the German Medical Association (article B.III. § 15.1) neither informed consent nor approval of an ethics committee was needed. The Ethics Committee of Hannover Medical School approved of this retrospective study (approval decision number 2375–2014). Patients provided informed consent that their data may be used for scientific purposes at the time of hospital admission which is the general policy of our institution. Patient records and patient data were anonymized and de-identified prior to analysis. None of the transplant donors were from a vulnerable population and all donors or next of kin provided written informed consent that was freely given.

### Clinical and demographic characteristics

1655 patients (983 males (59.4%) and 672 females (40.6%)) with a mean age of 50.0 years at transplantation (median 52.3 years, range: 8.4–76.4 years) met the inclusion and exclusion criteria. 40 patients (2.4%) were younger than 17 years at transplantation and classified as pediatric patients due to the application of different donor organ allocation rules until the age of 17. Indications for renal transplantation are summarized in S2 Table.

### Statistical analysis

Missing data was below 5% for all examined variables. Cases with missing data for variables examined in univariable and multivariable Cox regression were excluded.

Site-specific cancer incidence rates in the cohort were compared to the general German population using SIRs (SIR = observed incidence/expected incidence) with 95%-confidence-intervals (95%-CI) as described before [37]. Publicly available data for comparison were provided by the Robert-Koch-Institute, Berlin [38] and for tumor grading, T-stages and UICC-stages by the local state cancer registry (Niedersächsisches Krebsregister).

Univariable and risk-adjusted multivariable Cox regression analyses were used to determine independent pre-transplant risk factors for cancer-free survival. Cancer-free survival was defined as survival until death or the first diagnosis of de novo malignancy and measured until whichever incident was noted earlier.

After exclusion of multicollinearity in principal component analysis, variables with p-values ≤0.200 in univariable Cox regression analysis were included into an initial multivariable Cox regression model deploying purposeful variable selection. After the exclusion of significant two-way variable interactions, only those co-variables in the most parsimonious model were considered for inclusion into the preliminary Cox regression model, using a stepwise backward elimination method [39].

Assessment of geometrical distribution of the probability of malignancy diagnosis dates was carried out under the assumption that the number of opportunities for cancer detection was fairly stable using the probability density function (p = 0.028). This assumption appeared justified due to the relatively stable amount of annually performed transplants (mean 127 transplants, standard deviation 12.7 transplants with a range of 109–150 transplants per year).

**Fig 1:**
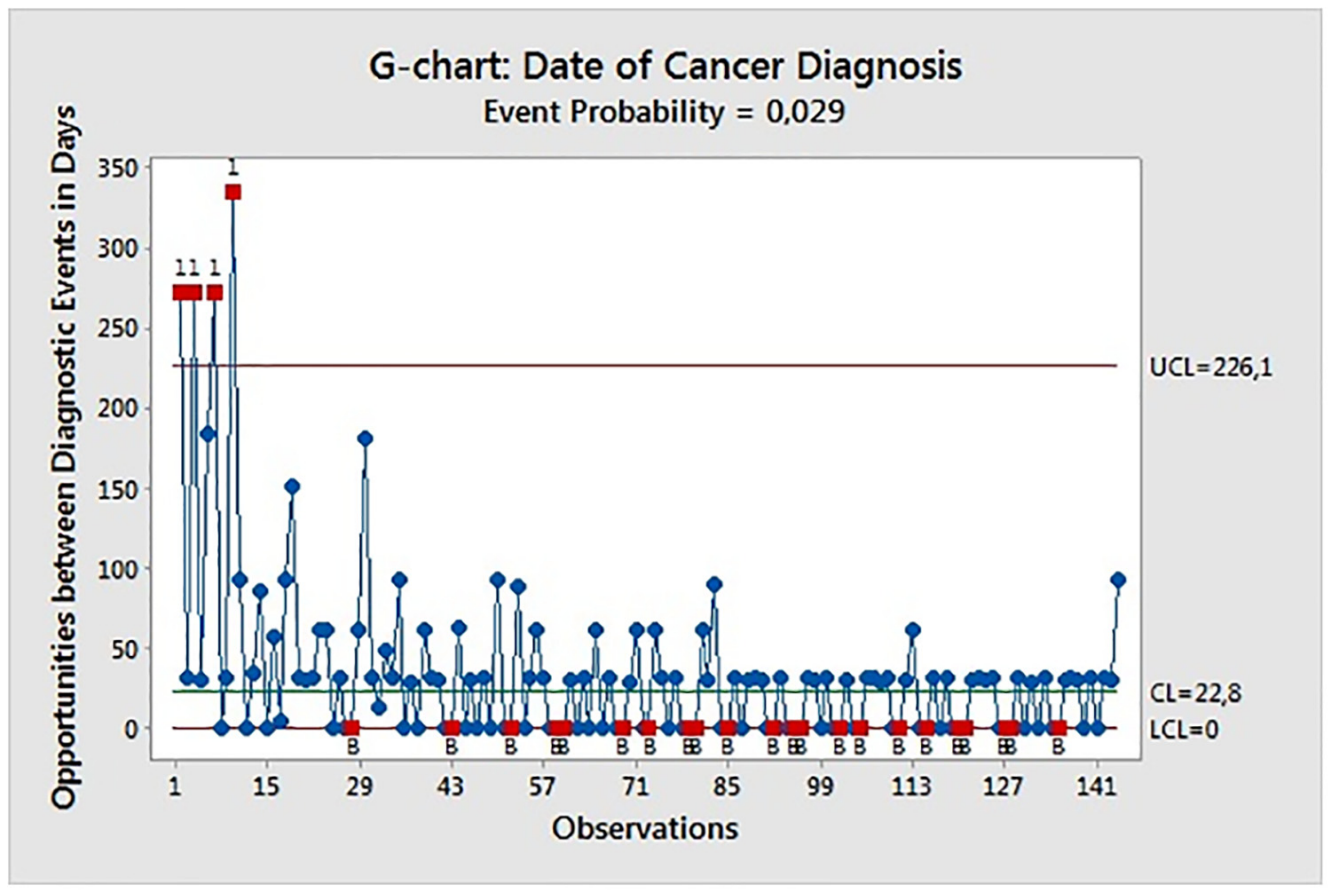
G-chart of Diagnosis Dates of Cancer. Cases marked with red dots and the number “1” above the upper control limit (UCL) indicate an unusually low rate of detected de novo malignancies (Test 1 result) which may raise concerns in regard to appropriate vigilance of doctors or possible decreased diagnostic sensitivity. Alternatively, this observation may also indicate decreased de novo cancer risk in these observed cases. Cases marked by the letter “B” and a red dot on the lower control limit (LCL) indicate a failed Benneyan test pointing to statistically relevant increases in the diagnostic event rate which should trigger clinical concerns in regard to causation, e.g. by intensified or uncontrolled immunosuppression. Alternatively, a more rigorous screening for cancer may also lead to an increased event rate.

G-chart analysis was used to illustrate the time at risk in days between consecutive dates of actual observations of post-transplant malignancy. The center line (CL), the upper control limit (UCL) and the lower control limit (LCL) were calculated and defined to demonstrate the central tendency and the range of natural variation of the plotted values [33, 34, 36]. The goal was the detection of values that fall outside the control limits and thereby exceed their statistical probability which indicates an unusually high increase or decrease in diagnostic frequency [33, 34, 36] (Fig 1).

The application of probability-based control limits reduced the false alarm rate and improved the capability to detect process changes [33, 34, 36].

The Benneyan test was deployed to identify a statistically relevant number of values below or at the LCL set to zero. Test 1 was deployed to detect relevant decreases in diagnostic event rates when times between events exceed three standard deviations with a probability of being above the UCL < = 0.0013499 [33, 34, 36]. This probability was used as the alpha value for the Benneyan test, which sets the probability of an alarm signal for a relevant increase in the event rate at the LCL < = 0.0013499. Test 1, by itself, has adequate power to detect a decrease but not an increase in the adverse event rate [33, 34, 36]. The use of the Benneyan test in combination with Test 1 provides the most significant improvement to detect increases in the adverse event rate [33, 34, 36].

Two-sided Chi^2^, Mann-Whitney-U tests and logistic regression were used where appropriate. For all statistical tests a p-value <0.05 was defined as significant. The statistical software packages Minitab 17 (Minitab Inc., State College, Pennsylvania, USA) and JMP version 11.0 (SAS Institute, Cary, North Carolina, USA) were used.

## Results

### Incidence and survival

152 de novo malignancies were observed in 144 patients (87 males and 57 females) (overall incidence 9.2%). The cumulative incidences of malignancies 5 and 10 years after transplantation were 5.2% and 8.2%, respectively. The SIR for all de novo malignancies was significantly increased (SIR = 2.75) (Table 1). S2 Table summarizes 5-year survival rates (Kaplan-Meier analysis) for patients after diagnosis of de novo malignancy after kidney transplantation with significantly increased SIRs in the current study in comparison to 5-year survival rates of cancer patients reported for the general German population.

**Table 1:** Standardized incidence ratios of post-transplant cancer types.

Diagnosis	Sex	Patients at risk	Observed cancers	Expected cancers	SIR	95% CI
**All neoplasms**	M+F	1655	152	55.26	**2.75**	**2.33–3.21**
	M	983	92	35.06	**2.62**	**2.12–3.19**
	F	672	60	20.20	**2.97**	**2.27–3.77**
**Pancreatic cancer**	M+F	1655	1	1.56	0.64	0.02–3.57
	M	983	1	1.05	0.95	0.02–5.3
	F	672	0	0.51	0	n.a.
**Lung cancer**	M+F	1655	10	6.77	1.57	0.75–2.89
	M	983	8	5.22	1.53	0.66–3.01
	F	672	2	1.55	1.29	0.16–4.66
**Colorectal cancer**	M+F	1655	6	6.85	0.88	0.32–1.91
	M	983	3	4.70	0.64	0.13–1.87
	F	672	3	2.15	1.39	0.29–4.06
**PTLD/NHL**	M+F	1655	14	1.67	**8.36**	**4.57–14.03**
	M	983	7	1.07	**6.54**	**2.63–13.47**
	F	672	7	0.60	**11.60**	**4.66–23.90**
**Gastric cancer**	M+F	1655	1	1.67	0.60	0.02–3.34
	M	983	0	1.23	0	n.a.
	F	672	1	0.44	2.28	0.06–12.69
**Esophageal cancer**	M+F	1655	3	0.92	3.57	0.73–10.43
	M	983	2	0.79	2.53	0.31–9.16
	F	672	1	0.13	7.96	0.20–44.33
**Renal cell carcinoma**	M+F	1655	40	1.87	**22.46**	**15.28–28.53**
	M	983	21	1.39	**15.08**	**9.32–22.22**
	F	672	19	0.48	**39.37**	**23.70–61.47**
**Bladder cancer**	M+F	1655	5	1.54	**3.24**	**1.05–7.57**
	M	983	4	1.28	3.11	0.85–7.97
	F	672	1	0.26	3.89	0.10–21.70
**Thyroid cancer**	M+F	1655	7	0.64	**10.13**	**4.07–20.87**
	M	983	0	0.24	0	n.a.
	F	672	7	0.40	**17.47**	**7.03–36.00**
**Malignant Melanoma**	M+F	1655	6	1.95	**3.08**	**1.13–6.71**
	M	983	3	1.14	2.64	0.54–7.71
	F	672	3	0.81	3.71	0.76–10.84
**Larynx cancer**	M+F	1655	1	0.68	1.46	0.04–8.14
	M	983	1	0.62	1.61	0.04–8.98
	F	672	0	0.06	0	n.a.
**Brain tumor**	M+F	1655	1	0.83	1.21	0.03–6.77
	M	983	0	0.55	0	n.a.
	F	672	1	0.28	3.61	0.09–20.10
**Leukemia**	M+F	1655	2	1.25	1.59	0.04–8.88
	M	983	1	0.86	1.16	0.03–6.47
	F	672	1	0.39	2.54	0.06–14.17
**Breast cancer**	M+F	1655	10	7.56	1.13	0.54–2.08
	M	983	1	0.08	12.87	0.33–71.71
	F	672	9	7.49	1.20	0.55–2.28
**Hepatocellular Carcinoma**	M+F	1655	4	0.92	**4.35**	**1.19–11.15**
	M	983	4	0.76	**5.25**	**1.43–13.44**
	F	672	0	0.16	0	n.a.
**Urothelial Carcinoma of the Ureter**	M+F	1655	1	0.06	16.98	0.43–94.63
	M	983	1	0.05	20.82	0.53–116.00
	F	672	0	0.01	0	n.a.
**Adrenal Gland Tumor**	M+F	1655	1	0.02	**43.23**	**1.09–240.88**
	M	983	1	0.01	**72.23**	**1.83–402.46**
	F	672	0	0.01	0	
**Prostate cancer**	M	983	20	9.02	**2.22**	**1.35–3.42**
**Other**	M+F	1655	19	n.a.	n.a.	n.a.

Shown are the observed numbers of incident cancer cases in the investigated cohort and expected numbers of incident cancer cases in the general population according to the site of cancer as well as their SIR-values with their respective 95%-confidence intervals with and without gender-matching (M = males, F = females). SIR-values based on less than five cancer cases are to be viewed cautiously due to methodological limitations and were not taken into further investigation. Significant SIR-values as indicated by the SIR values and the respective 95%-confidence intervals are indicated with bold numbers (n.a. = not applicable).

### Renal cell carcinoma

The risk for renal cell carcinoma (RCC) was significantly increased after transplantation as compared to the general population (Table 1). 37 patients developed 40 RCC with three cancers appearing consecutively in both autochthonous kidneys. These cancers were counted as separate entities when they were diagnosed with an interval of at least 30 days. 37 cancers were observed in autochthonous kidneys and three within the renal graft. Mean observed survival after first diagnosis of RCC was 1.9 years (median 1.5 years, range: 0.1–6.4 years). Two of 37 patients with de novo RCC died (5.4%) 57 and 1311 days after diagnosis. The 5-year survival rate after diagnosis of RCC was 77.8% in transplant patients compared to 65–69% in the general population (S2 Table). Patients who received a kidney from a living donor had a RCC incidence of 0.5% (n = 2) versus 2.8% (n = 35) after deceased donation (p = 0.006, Chi^2^) and were on average 10 years younger without reaching statistical significance (p = 0.787, Mann-Whitney test).

### Prostate cancer

20 patients experienced prostate cancer. The risk for prostate cancer after transplantation was significantly increased as compared to the general population (Table 1). The mean observed survival of patients with de novo prostate cancer was 2.8 years (median: 2.3 years, range: 0.4–8.2 years). Two of the 20 patients (10%) with de novo prostate cancer died 310 and 535 days after diagnosis. The 5-year survival rate after diagnosis was 87.4% in transplant patients and 78.0% in the general population (S2 Table). 19 of 20 patients with de novo prostate cancer were 50 years or older at transplantation.

### Bladder cancer

Five patients experienced bladder cancer. The risk for bladder cancer was significantly increased as compared to the general population (Table 1). Patients who suffered from bladder cancer after kidney transplantation had a mean observed survival time of 2.1 years (median: 1.1 years, range: 0.4 to 7.0 years). The 5-year survival rate after diagnosis was 80% in transplant patients and 41–47% in the general population (S2 Table).

### Post-transplant lymphoproliferative disorder (PTLD) / Non-Hodgkin Lymphoma (NHL)

The risk for PTLD/NHL was significantly increased as compared to the risk for NHL in the general population (Table 1) with a trend towards a better 5-year survival rate after diagnosis in transplanted patients (S2 Table). Patients who developed PTLD had a mean observed survival after diagnosis of 3.6 years (median: 1.1 years, range: 0 to 12.8 years). Four of 14 patients (28.6%) died within 9 to 438 days after diagnosis. 3 of these 4 patients (75%) were diagnosed with primary cerebral PTLD. Nine out of 14 cases (64.3%) with PTLD were 30 years or younger at transplantation. Five out of 14 cases (35.7%) with PTLD were transplanted during childhood (age <17 years). Three patients aged 30 years or younger died within 9 to 438 days after tumor diagnosis.

### Thyroid cancer

The risk for thyroid cancer after transplantation was significantly increased as compared to the general population (Table 1). All seven cases with de novo thyroid cancer were female patients (one case with follicular-oncocytic thyroid cancer and six cases with papillary thyroid cancer). All seven thyroid cancer patients survived during follow-up. For the general population 5-year survival rates between 82% and 89% have been reported (S2 Table).

### Malignant Melanoma

The risk for malignant melanoma was significantly increased (Table 1). The mean observed survival of patients with de novo melanoma was 3.3 years (median: 1.5 years, range: 0.1 to 11.2 years). Two of six patients died 508 and 518 days after diagnosis, respectively. Both cases were men with advanced T4 tumor stage at diagnosis (Table 2). The 5-year survival rate after diagnosis was 60% after transplantation and 78–86% in the general population (S2 Table).

**Table 2:** T-stages and Grading.

Cancer sites		Local cancer registry data	De novo malignancies after RTX		
**Prostate Cancer**			**n = 20**	UICC-stages not available	
Grading	**G1**	3.5%	0%		
	**G2**	41.7%	40%		
	**G3**	0%	10%		
	**G4**	43.6%	0%		
	**G9**	11.2%	0%		
T-stages	**1**	21.1%	40%		
	**2**	41.9%	15%		
	**3**	11.9%	0%		
	**4**	1.8%	0%		
	**x**	23.2%	45%		
**Melanoma**			**n = 6**	UICC-stages not available	
T-stages	**1**	63.3%	0%		
	**2**	11.5%	16.7%		
	**3**	7.3%	0%		
	**4**	6.3%	50%		
	**x**	11.6%	33.3%		
**Renal cell carcinoma**			**n = 40**	**UICC-**stages:	
Grading	**G1**	17.0%	25%		
	**G2**	54.5%	50%		
	**G3**	0.0%	25%		
	**G3-4**	18.2%	0%		
	**G9**	10.3%	0%		
T-stages	**1**	51.1%	85%	**0**	0%
	**2**	10.9%	2.5%	**I**	85%
	**3**	23.0%	12.5%	**II**	2.5%
	**4**	3.5%	0%	**III**	12.5%
	**x**	11.5%	0%	**IV**	0%
**Thyroid Cancer**			**n = 7**	**UICC**-stages:	
T-stages	**1**	46.5%	71.4%	**0**	0%
	**2**	13.6%	28.6%	**I**	57.1%
	**3**	15.9%	0%	**II**	14.3%
	**4**	3.9%	0%	**III**	28.6%
	**x**	20.1%	0%	**IV**	0%
**Bladder Cancer**			**n = 5**	UICC-stages not available	
T-stages	**is**	1.4%	0%		
	**a**	44.2%	0%		
	**1**	17.2%	80%		
	**2**	16%	20%		
	**3**	6.9%	0%		
	**4**	3.2%	0%		
	**x**	11.1%	0%		

Shown are the distributions of grading, T- and UICC-stages according to the different cancer sites with significantly increased SIRs in the current study. T-stages and grading were compared to the general population (data from the local cancer registry: Vertrauensstelle des niedersächischen Krebsregisters) (G9 = grading not classifiable, RTX = renal transplantation). For clarity and brevity only those cancer sites with a frequency ≥ 5 cases in this series and available comparative data on the local general population are shown. Grading data for prostate cancer was available for 50% of the study population with prostate cancer. All other prostate cancers were classified according to Gleason.

### Other de novo malignancies

Table 1 summarizes the patients at risk, the observed and expected cancer rates as well as the respective SIRs. Cancer-site specific SIR calculations based on less than five observed cases are unable to provide reliable results for comparison due to widely acknowledged methodological limitations.

### Possible role of the underlying kidney disease

Autosomal dominant polycystic kidney disease (ADPKD), polycystic kidney disease other than ADPKD (PKD), and diabetic nephropathy were significantly associated with cancer-free survival after kidney transplantation in univariable Cox regression analysis (Table 3). Transplant indications with less than 25 cases in the study population were not analyzed in univariable Cox regression.

**Table 3:** Distribution of de novo malignancy and univariable Cox regression model.

Variables		Distribution		p-value*	univariable Cox regression		
		No cancer	De novo cancer		p-value	HR	95% -CI
**Patient characteristics**	Age at transplant in years	Mean: 49.6 (SD: 14.9)	Mean: 54.6 (SD: 14.6)	0.001	<0.001	1.012	1.008–1.016
	Age at transplant <17 years (pediatric)	n = 35(87.50%)	n = 5(12.50%)	0.388	0.001	0.595	0.418–0.819
	**Age at transplant 1**^**st**^ **Quartile (<39.9 years)**	**n = 394(95.86%)**	**n = 17(4.14%)**	**0.0002**	**0.001**	**0.822**	**0.732–0.922**
	**Age at transplant 2**^**nd**^ **Quartile (39.91–52.25 years)**	**n = 381(91.59%)**	**n = 35(8.41%)**	**0.810**	**<0.001**	**0.801**	**0.712–0.899**
	Age at transplant 3^rd^ Quartile (52.26–62.61 years)	n = 375(90.36%)	n = 40(9.64%)	0.434	0.117	1.099	0.976–1.234
	**Age at transplant 4**^**th**^ **Quartile (>62.61 years)**	**n = 361(87.41%)**	**n = 52(12.59%)**	**0.001**	**<0.001**	**1.515**	**1.342–1.707**
	Gender (male/female)	n.a.			0.066	1.101	0.994–1.220
	Male gender	n = 896(91.15%)	n = 87(8.85%)	0.794	n.a.		
	Female gender	n = 615(91.52%)	n = 57(8.48%)	0.794			
	**Body mass index in kg/m**^**2**^	**Mean: 25.0 (SD: 4.0)**	**Mean: 25.3 (SD: 3.6)**	**0.437**	**<0.001**	**1.042**	**1.028–1.055**
	Body mass index > 30 kg/m^2^	n = 177(93.16%)	n = 13(6.84%)	0.333	<0.001	1.600	1.362–1.868
	Diabetes	n = 297(91.38%)	n = 28(8.62%)	0.951	0.012	1.181	1.038–1.339
	Diabetes type 1	n = 19(90.48%)	n = 2(9.52%)	0.893	0.425	1.209	0.741–1.884
	Diabetes type 2	n = 278(91.45%)	n = 26(8.55%)	0.919	0.018	1.173	1.028–1.335
	**Insulin dependent diabetes**	**n = 170(92.39%)**	**n = 14(7.61%)**	**0.577**	**0.039**	**1.188**	**1.008–1.389**
	Smoking history	n = 208(89.27%)	n = 25(10.73%)	0.236	0.367	0.935	0.805–1.080
	Smoking and consecutive COPD	n = 70(87.50%)	n = 10(12.50%)	0.217	0.349	1.124	0.876–1.417
	Duration of pre TX dialysis in years	Mean: 5.4 (SD: 4.1)	Mean: 5.9 (SD: 2.9)	0.154	0.861	1.001	0.986–1.014
	**Pre-TX malignoma**	**n = 98(85.22%)**	**n = 17(14.78%)**	**0.016**	**0.048**	**1.237**	**1.002–1.510**
**Transplant indications**	IgA nephropathy	n = 189(91.30%)	n = 18(8.70%)	0.998	0.972	0.997	0.854–1.158
	**Autosomal dominant polycystic kidney disease (ADPKD)**	**n = 177(96.20%)**	**n = 7(3.80%)**	**0.012**	**<0.001**	**1.393**	**1.186–1.625**
	Chronic glomerulonephritis	n = 166(90.71%)	n = 17(9.29%)	0.765	0.444	0.939	0.797–1.100
	Benign nephrosclerosis	n = 118(88.72%)	n = 15(11.28%)	0.272	0.620	1.049	0.865–1.260
	**Diabetic nephropathy**	**n = 84(92.31%)**	**n = 7(7.69%)**	**0.726**	**0.001**	**1.519**	**1.209–1.882**
	**Polycystic kidney disease other than ADPKD**	**n = 71(91.03%)**	**n = 7(8.97%)**	**0.930**	**0.003**	**0.709**	**0.553–0.893**
	Focal segmental glomerulosclerosis	n = 48(90.57%)	n = 5(9.43%)	0.847	0.775	0.959	0.710–1.263
	Reflux nephropathy	n = 49(98.0%)	n = 1(2.0%)	0.088	0.089	1.293	0.960–1.699
	Nephrocirrhosis	n = 44(95.65%)	n = 2(4.35%)	0.288	0.677	1.067	0.779–1.421
	Mesangial proliferative glomerulonephritis	n = 41(91.11%)	n = 4(8.89%)	0.964	0.083	0.730	0.526–0.983
	Interstitial nephritis	n = 39(97.50%)	n = 1(2.50%)	0.159	0.614	0.922	0.660–1.249
	Membranoproliferative glomerulonephritis	n = 23(88.46%)	n = 3(11.54%)	0.605	0.096	1.450	0.932–2.135
	Alport syndrome	n = 28(90.32%)	n = 3(9.68%)	0.846	0.856	1.035	0.695–1.474
	Vascular nephropathy	n = 33(97.06%)	n = 1(2.94%)	0.229	0.061	1.417	0.983–1.966
	**Other indications**	**n = 401(88.33%)**	**n = 53(11.67%)**	**0.008**	**0.003**	**0.843**	**0.751–0.944**

Shown are the results of univariable Cox regression analysis to determine pre-transplant risk factors for the development of post-transplant de novo malignancy during follow-up (HR = hazard ratio, 95%-CI = 95% confidence interval, p-values were determined with the effect likelihood ratio test) and the distribution of diagnostic events of malignancy within the study population (n.a. = not applicable; SD = standard deviation). P-values* for the distribution were determined with the Chi^2^ test for binary variables and with the Mann-Whitney-U test for continuous variables. Transplant indications with 25 or less cases in the study population were summarized as “other indications” for the purpose of Cox regression modelling.

### Other pre-transplant variables as risk factors

141 out of 1555 patients undergoing dialysis prior to transplantation developed post-transplant de novo malignancies (9.07%, p-value = 0.0369). The duration of pre-transplant dialysis did not significantly influence the risk of cancer development during follow-up (Table 3). Preemptive transplantation without prior dialysis was carried out predominantly in young patients. Only three patients with preemptive transplantation developed de novo malignancy. All three cases were pediatric patients (age 9.7–15.2 years at transplantation) who developed PTLD 2.6–9.7 years after transplantation.

17 out of 411 patients transplanted under the age of 39.9 years (1st quartile) presented with post-transplant de novo cancer while the incidence of malignancy was higher in the age group older than 62.6 years at transplantation (4th quartile). Age groups in quartiles demonstrated a differentiated influence on cancer free-survival (Table 3).

17 out of 115 patients with a history of cancer prior to transplantation developed de novo malignancies (Table 3). There were no post-transplant recurrences of the same cancer-type in those patients with pre-transplant malignancies. The choice of treatment of pre-transplant malignancies in terms of chemotherapy or radiotherapy did not significantly influence the development of de novo malignancies (data not shown).

After living-related kidney transplantation 22 out of 409 patients developed de novo cancer during follow-up (5.38%, p-value = 0.006, Chi^2^). Furthermore, univariable Cox regression analysis revealed a significant influence of body mass index and insulin-dependent diabetes on cancer-free survival (Table 3).

### Independent risk factors for cancer-free survival

Risk-adjusted multivariable Cox regression revealed that age below 52.3 years at transplantation and the indication PKD other than ADPKD were significant independent protective factors while age at transplantation older than 62.6 years, high body mass index, ADPKD and diabetic nephropathy were independent risk factors for cancer-free survival (Table 4).

**Table 4:** Multivariable Cox regression model and G-chart alarm signals.

Variables	Multivariable Cox regression			G-chart alarm signals			
	p-value	HR	95% -CI	Cases with alarm signals above UCL	p-value	Cases with alarm signals on LCL	p-value
Age 1^st^ Quartile (<39.90 years)	0.103	0.882	0.759–1.026	1 (6.3%)	0.374*	2 (12.4%)	0.734*
**Age 2**^**nd**^ **Quartile (39.91–52.25 years)**	**0.007**	**0.819**	**0.708–0.946**	0 (0%)	0.248*	6 (17.1%)	0.740*
**Age 4**^**th**^ **Quartile (>62.61 years)**	**0.001**	**1.289**	**1.114–1.492**	2 (3.9%)	0.565*	8 (15.4%)	1.000*
**Body mass index in kg/m**^**2**^	**<0.001**	**1.036**	**1.022–1.050**	Median: 23.2 (range: 23.1–25.3)	0.348**	Median: 25.5 (range: 20.2–32.5)	0.443**
Insulin dependent diabetes	0.298	0.903	0.740–1.093	0 (0%)	0.504*	4 (28.6%)	0.150*
Pre-TX malignoma	0.235	1.137	0.918–1.392	0 (0%)	0.456*	4 (25.5%)	0.321*
**Autosomal dominant polycystic kidney disease (ADPKD)**	**0.008**	**1.262**	**1.065–1.489**	0 (0%)	0.645*	2 (28.6%)	0.321*
**Diabetic nephropathy**	**0.004**	**1.506**	**1.145–1.963**	0 (0%)	0.645*	1 (14.3%)	0.934*
**Polycystic kidney disease other than ADPKD**	**0.001**	**0.679**	**0.527–0.862**	0 (0%)	0.645*	2 (28.6%)	0.321*
**Other indications**	**0.044**	**0.883**	**0.781–0.997**	2 (3.8%)	0.587*	5 (9.4%)	0.130*

Shown are the results of risk-adjusted multivariable Cox regression analysis to determine independent pre-transplant risk factors for the development of post-transplant de novo malignancy during follow-up (HR = hazard ratio, 95%-CI = 95% confidence interval, p-values were determined with the effect likelihood ratio test). Further shown is the statistical significance of the differences of distributions of investigated variables for cases with alarm signals above the upper control limit (UCL) and cases with alarm signals on the lower control limit (LCL) versus all other cases, respectively (* Chi^2^ test, ** logistic regression analysis).

### G-chart analysis

G-chart analysis revealed four cases with de novo malignancy above the upper control limit (UCL) indicating an unusually low rate of detected de novo malignancies. 22 cases failed the Benneyan test indicating relevant increases in de novo malignancy detection rates (Fig 1). G-chart analysis identified relevant changes in the detection rates of cancer during aftercare with no significant relation to identified risk factors for cancer-free survival (p<0.05) (Table 4).

## Discussion

This study is unique as it provides the distribution of tumor stages of de novo malignancies in comparison to cancers in the general population and identifies underlying causes of end-stage renal failure as independent risk factors for cancer-free survival.

This is the first study that reports SIRs in kidney transplant recipients from Germany with additional analysis of independent risk factors for cancer-free survival. These incidence rates were almost three times as high as compared to the age- and sex-matched general population (SIR 2.75). Higher incidence rates have been reported from Australia and New Zealand (SIR 3.27) [24] indicating that cancer incidence rates after kidney transplantation vary substantially in different geographic regions.

As in previous studies with non-melanoma skin cancer excluded, RCC was the most commonly observed cancer entity. The SIR of RCC was higher in this study (Table 1) as compared to previous reports [23, 25, 26, 28–29]. This higher incidence may be due to more systematic and rigorous screening for RCC in the investigated cohort. The fact that tumor stages were lower in transplanted patients as compared to the matched general population lends weight to this notion (Table 2).

In contrast to previous studies [20, 40–41], the smoking history or the duration of dialysis prior to transplantation did not significantly influence the development of renal cell cancer. Unfortunately, the smoking history is not well documented in many patient files lending doubt to the assessment of this widely recognized risk factor.

Surprisingly and in clear contrast to our previously published observations concerning liver transplantation [32], vulva cancer was not observed after kidney transplantation in this cohort. Vulva cancer is associated with the occurrence of human papillomavirus [42] which is more common in immunosuppressed patients [43, 44]. One would expect the incidence to be much higher since a more intense immunosuppressive therapy is typically applied after renal transplantation. This observation may point to a lack of systematic gynecological follow-up in kidney transplant patients as compared to liver transplant patients in our institution with different doctors in charge for aftercare.

Patients with de novo malignancies were approximately 10 years younger in this study than patients with comparable malignancies in the general population (S3 Table). Frequently, de novo malignancies were diagnosed at an earlier tumor stage with a tendency towards lower grading (Table 2). This may be due to more frequent and thorough cancer surveillance in transplanted patients. The more intense surveillance may have led to an overestimation of SIRs. The size of this bias is unclear, but unlikely to reduce the cancer risk to the level of the general population, because 10 years lead time (time period of earlier diagnosis) is not described in cancer. As a consequence, patients who experienced de novo malignancy with a lower tumor stage had a slightly better 5-year survival rate (except melanoma) than the general population with the same cancer entities (S2 Table). This finding may also be explained by the fact that younger patients are more likely to tolerate more aggressive cancer treatments than older patients. Moreover, cancer treatments in transplant patients are mostly conducted by the tertiary referral center of the transplant institution which can be expected to provide a maximum of therapeutic resources. The comparatively worse 5-year survival rate of melanoma in transplant patients is probably due to advanced tumor stages at diagnosis (Table 2), showing room for improvement in our post-transplant melanoma screening approach. However, the low number of patients diagnosed with malignant melanoma may have biased this finding.

Body mass index was identified here as an independent risk factor in multivariable analysis. Obesity has been reported to promote tumor growth in non-transplanted patients [45]. Several factors such as increased levels of growth factors and steroid hormones, a change in adipocytokine levels and a constant low-grade inflammation contributing to the liberation of growth-promoting cytokines and immune modulation are held responsible for this connection [45].

Furthermore, age was identified as independent risk factor for cancer development after transplantation. Various factors such as the degeneration of DNA, mutations, aberrant replication and apoptosis leading to an increased tumor growth have been identified in previous studies, independently of kidney transplantation [46, 47].

Interestingly, PKD was identified as an independent protective factor while ADPKD was an independent risk factor for cancer-free survival. A very recent study also described a lower cancer incidence in renal transplant patients with PKD as compared to transplanted patients without PKD [48]. The reason for this finding is not understood so far and might be related to biologic characteristics of PKD or to cancer risk behaviors associated with PKD including pre-transplant nephrectomy. On the other hand and in contrast to previous studies, ADPKD was associated with a higher risk of cancer development after transplantation [49]. This observation is explained by the fact that patients with ADPKD in this study developed malignancies at an earlier time point during follow-up as compared to patients with PKD (3.61 years vs. 6.057 years) as identified with Cox regression which considers time to event data. Furthermore, this discrepancy might also be due to a principal classification and diagnosis problem since not every patient with kidney cysts is genetically tested for ADPKD.

Diabetic nephropathy emerged as an independent risk factor for cancer-free survival. Diabetes per se is known to promote tumor-growth [50]. This observation and the fact that insulin-dependent diabetes and diabetic nephropathy show some collinearity likely explains why insulin-dependent diabetes could not be identified as an independent risk factor in multivariable Cox regression. This observation might point to a time-dependency of cancer development in patients suffering from insulin-dependent diabetes.

Due to frequent changes in dosages and types of immunosuppressive medication during follow-up it was not possible to analyze the impact of the complete immunosuppressive therapy on de novo cancer risk systematically. We believe that an analysis of the complete immunosuppressive medication over time which takes into account all changes of dosages, medication and immunosuppressants’ blood levels would be required.

Pre-transplant risk factors for de novo cancer have not been well described before while post-transplant risk factors for de novo cancer such as ATG induction, mTOR inhibitors, rejection and EBV status are already known and have been described in several previous publications. The definition of post-transplant risk factors for the development of de novo cancer after kidney transplantation was not the goal of the current study. The main goal of the current study was to explore the potential of G-chart analysis for process improvement and benchmarking of post-transplant cancer surveillance during ongoing post-transplant management. The independent pre-transplant risk factors described in this study help to inform the post-transplant management process prior to and immediately after transplantation. These pre-transplant risk factors further inform the interpretation of ongoing prospective G-charts with the aim to improve post-transplant process control as early as possible. The identified pre-transplant risk factors may help to adapt clinical post-transplant management individually for patients with pre-transplant risk factors for de novo cancer, e.g. by choosing different types and intensities of immunosuppression.

We believe that the results of this retrospective study show the principal value of G-chart analysis for the early identification of relevant increases and decreases in de-novo malignancy detection rates. Several advantages of G-charts in comparison to standard Shewhart-charts need to be addressed. First, G-charts are able to provide useful results even if the availability or frequency of the plotted values is low, which is the case for de-novo malignancies. Conventional charts such as n, np or c-charts will need a certain number of values to detect a significant process change. This can necessitate waiting until the end of longer time periods to calculate and interpret the results [33]. In such manner it would not be possible to intervene in real-time. Second, G-charts are easy to use and interpret, so these charts could be updated in outpatient departments without the need of a statistician. Third, to analyze “number- or cases-between-events-type” of data, none of the standard charts are appropriate, because this kind of data most likely meets the conditions of a geometric distribution. The use of other than G-charts may result in wrong conclusions.

Prospective deployment of G-chart analysis may identify alarm signals in case of significantly decreased or increased cancer detection rates. This allows early root cause analysis, for example by conducting a Kaizen event and additional statistical analyses including re-evaluation of independent risk factors for cancer-free survival. For a Kaizen event clinical managers and process owners as well as transplant experts are gathered to map the current process in a deployment flow chart looking for process improvements and possible root causes for unusually high or low defect rates [51]. Varying effectiveness of cancer surveillance schemes, inconsistent alertness to potential cancer symptoms by doctors and/or patients, new immunosuppressive protocols or uncontrolled immunosuppression, epidemiological changes of cancer risks and the introduction of more or less sensitive screening methods may all be possible root causes for observed relevant changes in cancer detection rates.

Prospective G-charts could be used to facilitate visual management in outpatient clinics with the goal to increase the awareness and alertness in regard to cancer risks after transplantation. This may ultimately lead to improved cancer detection or even prevention in transplant patients [33, 34]. As has been suggested by Benneyan, adjustment of G-charts to the number of patients at risk likely renders this tool more effective [33, 34]. We believe that risk-adapted cancer surveillance combined with prospective G-chart analysis can improve cancer surveillance because G-chart alarms provide additional information about the quality of cancer surveillance calling for root-cause analysis to improve the surveillance process. Comparative G-chart analysis may enable benchmarking of cancer surveillance protocols. Transplant registries should collect data on detected de novo malignancies and implement G-chart analysis in order to facilitate transplant center benchmarking to assess and compare the quality of cancer surveillance schemes.

We accept the methodological limitations of the current study including the lack of timely root cause analysis for the G-chart findings which is owed to the retrospective nature of this study. This also implies that there is a possibility of underreporting of cancer. Further limitations of this study are caused by the small sample size of some cancer types and primary diseases and the fact that non-melanoma skin-cancer, the most common form of cancer in transplanted patients, were not analyzed. Furthermore, a likely center bias has to be taken into account. Altogether, we believe that this study clearly demonstrates a powerful methodological approach to the improvement of cancer surveillance management in transplant aftercare at the transplant center level providing a perspective for transplant center benchmarking.

## Conclusions

This study determined site-specific cancer incidence rates after kidney transplantation, showing that de-novo malignancies are a current and important issue to be addressed. Furthermore, this study showed that certain pre-transplant characteristics play an important role in terms of de-novo malignancies after transplantation. The usage of G-chart analysis during post-transplant management can improve cancer surveillance processes by triggering Kaizen events.

## Supporting Information

S1 Table Indications for renal transplantation. Shown are the causes of end-stage renal failure with ≥ 10 cases leading to kidney transplantation in adult and pediatric recipients of the study-population.
(DOCX)

S2 Table 5-year survival rates. Shown are the 5-year survival rates (Kaplan-Meier analysis) for patients after diagnosis of de novo malignancy after kidney transplantation with significantly increased SIRs in the current study in comparison to 5-year survival rates of cancer patients reported for the general German population by the Robert Koch-Institute, Berlin in 2010 (n.a. = not applicable).
(DOCX)

S3 Table Mean age at diagnosis of cancer. Shown is the time interval between renal transplantation (RTX) and the diagnosis of different de novo malignancies with significantly elevated SIRs in years and the mean patient ages at the time of diagnosis of cancer in the general population and after kidney transplantation. * Brackets show mean ages at diagnosis of in situ tumor stages and neoplasms with unknown or uncertain characteristics. (n.a. = not applicable, RKI = Robert Koch-Institute).
(DOCX)
