# Supplementary material for: Independent Pre-Transplant Recipient Cancer Risk Factors after Kidney Transplantation and the Utility of G-Chart Analysis for Clinical Process Control
Source: PLoS One. 2016 Jul 11;11(7):e0158732. doi: 10.1371/journal.pone.0158732 (PMC4939933; doi:10.1371/journal.pone.0158732)
Supplement: S1 Table — Shown are the causes of end-stage renal failure with ≥ 10 cases leading to kidney transplantation in adult and pediatric recipients of the study-population. (DOCX) [file pone.0158732.s001.docx]

Supplementary Table 1: Indications for renal transplantation

| **Indications for RTX** | **Total** | **Adult recipients**  (age > 17 years) | | **Pediatric recipients**  (age < 17 years) | |
| --- | --- | --- | --- | --- | --- |
|  | **n** | **n** | **% of all adult recipients** | **n** | **% of all pediatric recipients** |
| IgA nephropathy | 207 | 207 | 12.8 | 0 | 0 |
| Autosomal dominant polycystic kidney disease (ADPKD) | 184 | 183 | 11.3 | 1 | 2.5 |
| Chronic glomerulonephritis | 183 | 183 | 11.3 | 0 | 0 |
| Benign nephrosclerosis | 133 | 133 | 8.2 | 0 | 0 |
| Chronic renal failure of unknown genesis | 93 | 93 | 5.8 | 0 | 0 |
| Diabetic nephropathy | 91 | 91 | 5.6 | 0 | 0 |
| Polycystic kidney disease other than ADPKD | 78 | 78 | 4.8 | 0 | 0 |
| Focal segmental glomerulosclerosis | 53 | 53 | 3.3 | 0 | 0 |
| Reflux nephropathy | 50 | 46 | 2.8 | 4 | 10.0 |
| Nephrocirrhosis | 46 | 46 | 2.8 | 0 | 0 |
| Mesangial proliferative glomerulonephritis | 45 | 45 | 2.8 | 0 | 0 |
| Interstitial nephritis | 40 | 39 | 2.4 | 1 | 2.5 |
| Vascular nephropathy | 34 | 34 | 2.1 | 0 | 0 |
| Alport syndrome | 31 | 30 | 1.9 | 1 | 2.5 |
| Membranoproliferative glomerulonephritis | 26 | 26 | 1.6 | 0 | 0 |
| Analgesic nephropathy | 25 | 25 | 1.5 | 0 | 0 |
| Wegener’s granulomatosis | 24 | 23 | 1.4 | 1 | 2.5 |
| Obstructive uropathy | 20 | 16 | 1.0 | 4 | 10.0 |
| Nephronophtisis | 19 | 10 | 0.6 | 9 | 22.5 |
| Malignant nephrosclerosis | 16 | 16 | 1.0 | 0 | 0 |
| Rapidly progressive glomerulonephritis | 16 | 13 | 0.8 | 3 | 7.5 |
| Chronic Pyelonephritis | 15 | 15 | 0.9 | 0 | 0 |
| Membranous glomerulonephritis | 15 | 15 | 0.9 | 0 | 0 |
| Renal dysplasia | 15 | 8 | 0.5 | 7 | 17.5 |
| Nephrolithiasis | 14 | 14 | 0.9 | 0 | 0 |
| Minimal change disease | 12 | 12 | 0.7 | 0 | 0 |
| Amyloidosis | 11 | 11 | 0.7 | 0 | 0 |
| Goodpasture syndrome | 11 | 11 | 0.7 | 0 | 0 |
| Systemic lupus erythematosus | 11 | 11 | 0.7 | 0 | 0 |
| Hemolytic-uremic syndrome | 10 | 8 | 0.5 | 2 | 5.0 |
| Acute proliferative glomerulonephritis | 10 | 10 | 0.6 | 0 | 0 |
| Henoch-Schönlein Purpura | 10 | 9 | 0.6 | 1 | 2.5 |
| Other | 107 | 101 | 6.5 | 6 | 15 |
| **Total** | **1655** | **1615** | **100** | **40** | **100** |

Shown are the causes of end-stage renal failure with > 10 cases leading to kidney transplantation in adult and pediatric recipients of the study-population.
