# Supplementary material for: Independent Pre-Transplant Recipient Cancer Risk Factors after Kidney Transplantation and the Utility of G-Chart Analysis for Clinical Process Control
Source: PLoS One. 2016 Jul 11;11(7):e0158732. doi: 10.1371/journal.pone.0158732 (PMC4939933; doi:10.1371/journal.pone.0158732)
Supplement: S2 Table — Shown are the 5-year survival rates (Kaplan-Meier analysis) for patients after diagnosis of de novo malignancy after kidney transplantation with significantly increased SIRs in the current study in comparison to 5-year survival rates of cancer patients reported for the general German population by the Robert Koch-Institute, Berlin in 2010 (n.a. = not applicable). (DOCX) [file pone.0158732.s002.docx]

Supplementary Table 2: 5-year survival rates

| **Diagnosis** | **Patients**  **(n)** | **5-year survival in %** (Kaplan-Meier) | **RKI data for the general population:**  **5-year survival in %** | |
| --- | --- | --- | --- | --- |
|  |  |  | **Male** | **Female** |
| Renal cell carcinoma | 37 | 77.8 | 65 | 69 |
| Prostate Cancer | 20 | 87.4 | 78 | n.a. |
| Bladder Cancer | 5 | 80.0 | 47 | 41 |
| PTLD/ NHL | 14 | 67.3 | 57 | 59 |
| Thyroid cancer | 7 | 100.0 | 82 | 89 |
| Melanoma | 6 | 60.0 | 78 | 86 |

Shown are the 5-year survival rates (Kaplan-Meier analysis) for patients after diagnosis of de novo malignancy after kidney transplantation with significantly increased SIRs in the current study in comparison to 5-year survival rates of cancer patients reported for the general German population by the Robert Koch-Institute, Berlin in 2010 (n.a. = not applicable).
