# Supplementary material for: Independent Pre-Transplant Recipient Cancer Risk Factors after Kidney Transplantation and the Utility of G-Chart Analysis for Clinical Process Control
Source: PLoS One. 2016 Jul 11;11(7):e0158732. doi: 10.1371/journal.pone.0158732 (PMC4939933; doi:10.1371/journal.pone.0158732)
Supplement: S3 Table — Shown is the time interval between renal transplantation (RTX) and the diagnosis of different de novo malignancies with significantly elevated SIRs in years and the mean patient ages at the time of diagnosis of cancer in the general population and after kidney transplantation. * Brackets show mean ages at diagnosis of in situ tumor stages and neoplasms with unknown or uncertain characteristics. (n.a. = not applicable, RKI = Robert Koch-Institute). (DOCX) [file pone.0158732.s003.docx]

|  | | | **Mean age at diagnosis of cancer** | | | |
| --- | --- | --- | --- | --- | --- | --- |
|  |  |  | **General**  **Population (RKI)** | | **Post RTX** | |
| **Diagnosis** | **Cancers**  **(n)** | **Mean time in years**  **RTX - diagnosis** | **Males** | **Females** | **Males** | **Females** |
| All neoplasms | 152 | 4.4  (median 3.6; range 0.1-13.0) | 69 | 69 | 59.7 | 58.1 |
| Renal cell carcinoma | 40 | 3.2  (median 3.1; range 0.1-8.4) | 68 | 71 | 53.6 | 60.9 |
| Prostate Cancer | 20 | 3.2  (median 3.1; range 0.1-8.4) | 70 | n.a. | 66.7 | n.a. |
| Bladder cancer | 5 | 3.3  (median 2.8; range 0.2-7.7) | 73 (72)* | 77 (74)* | 61.0 | 70.7 |
| PTLD/NHL | 14 | 4.3  (median 2.6; range 0.5-9.7) | 69 | 71 | 34.4 | 32.8 |
| Thyroid cancer | 7 | 3.2  (median 2.5; range 0.1-8.2) | 56 | 52 | n.a. | 61.4 |
| Melanoma | 6 | 3.1  (median 3.5; range 0.2-5.5) | 66 | 58 | 65.2 | 53.0 |

Supplementary Table 3: Mean age at diagnosis of cancer

Shown is the time interval between renal transplantation (RTX) and the diagnosis of different de novo malignancies with significantly elevated SIRs in years and the mean patient ages at the time of diagnosis of cancer in the general population and after kidney transplantation. * Brackets show mean ages at diagnosis of in situ tumor stages and neoplasms with unknown or uncertain characteristics. (n.a. = not applicable, RKI = Robert Koch-Institute).
